# Mosquito Akirin as a potential antigen for malaria control

**DOI:** 10.1186/1475-2875-13-470

**Published:** 2014-12-03

**Authors:** Mário da Costa, Renato Pinheiro-Silva, Sandra Antunes, Juan A Moreno-Cid, Ana Custódio, Margarita Villar, Henrique Silveira, José de la Fuente, Ana Domingos

**Affiliations:** Instituto de Higiene e Medicina Tropical, Rua da Junqueira 100, 1349-008 Lisbon, Portugal; SaBio. Instituto de Investigación en Recursos Cinegéticos IREC, CSIC-UCLM-JCCM, Ronda de Toledo s/n, 13005 Ciudad Recal, Spain; Department of Veterinary Pathobiology, Center for Veterinary Health Sciences, Oklahoma State University, Stillwater, OK 74078 USA; Centro de Malária e Outras Doenças Tropicais, Instituto de Higiene e Medicina Tropical, Rua da Junqueira 100, 1349-008 Lisbon, Portugal

**Keywords:** Akirin, Arthropod, Mosquito, Subolesin, Vaccine, Malaria

## Abstract

**Background:**

The control of vector-borne diseases is important to improve human and animal health worldwide. Malaria is one of the world’s deadliest diseases and is caused by protozoan parasites of the genus *Plasmodium*, which are transmitted by *Anopheles* spp. mosquitoes. Recent evidences using Subolesin (SUB) and Akirin (AKR) vaccines showed a reduction in the survival and/or fertility of blood-sucking ectoparasite vectors and the infection with vector-borne pathogens. These experiments suggested the possibility of using AKR for malaria control.

**Methods:**

The role of AKR on *Plasmodium berghei* infection and on the fitness and reproduction of the main malaria vector, *Anopheles gambiae* was characterized by evaluating the effect of *akr* gene knockdown or vaccination with recombinant mosquito AKR on parasite infection levels, fertility and mortality of female mosquitoes.

**Results:**

Gene knockdown by RNA interference in mosquitoes suggested a role for *akr* in mosquito survival and fertility. Vaccination with recombinant *Aedes albopictus* AKR reduced parasite infection in mosquitoes fed on immunized mice when compared to controls.

**Conclusions:**

These results showed that recombinant AKR could be used to develop vaccines for malaria control. If effective, AKR-based vaccines could be used to immunize wildlife reservoir hosts and/or humans to reduce the risk of pathogen transmission. However, these vaccines need to be evaluated under field conditions to characterize their effect on vector populations and pathogen infection and transmission.

## Background

Malaria, one of the world’s deadliest diseases, is caused by protozoan parasites of the genus *Plasmodium* which are transmitted by *Anopheles* spp. mosquitoes [1]. Last malaria report from WHO, estimates that 3.4 billion people were at disease risk in 2013 [2]. *Plasmodium* spp. have a complex multi-stage life cycle involving two hosts, primary host (mosquito) and secondary host (human) occurring in different cellular environments [3].

Recently, methodologies for diagnosis and integrated vector control by various physical and chemicals methods have been improved or implemented and research to develop vaccines against malaria is being carried on by several groups around the world [3]. The development of a vaccine against malaria has been a difficult task, mainly due to the complexity of the parasite life cycle and the equally complex and multifaceted host immune responses [4]. Malaria vaccines targeting the blood stage are considered as anti-disease vaccines as they prevent or reduce clinical disease but do not prevent infection [4]. An efficacious pre-erythrocytic vaccine would block disease by inhibiting parasites to reach the blood stream and preventing transmission. The RTS,S/AS is an example of a candidate pre-erythrocytic vaccine at phase III field trials performed in eleven African research centres [5, 6]. However, the first results from these trials were not as good as expected [7]. Recently, another pre-erythrocytic candidate vaccine based on whole attenuated sporozoites has been developed; phase I trials were concluded showing several weaknesses such as the need for intravenous administration of very high number of sporozoites to achieve complete immune protection in vaccinated individuals [8]. This could be overcome by transmission-blocking vaccines that specifically intend to target molecules that are exclusive to gametocytes or to other mosquito stages. Antibodies against these targets are capable of blocking the development of parasite sexual stages and, therefore, interrupt transmission. A vaccine based on mosquito-stage proteins of both *Plasmodium falcip*arum and *Plasmodium vivax* was shown to produce dose-dependent antibody-mediated transmission-blocking activity but showed to be unacceptably reactogenic [9].

Preliminary results obtained in arthropod vectors with impact on human and animal health have shown that protective antigens may be used for the development of vaccines against both vectors and pathogens they transmit [10–18]. Among these antigen candidates, tick Subolesin (SUB) and the ortholog in insects, Akirin (AKR), have been used to induce a protective response in vaccinated hosts for the control of hard (*Ixodes* spp., *Rhipicephalu*s spp., *Amblyomma americanum*, *Dermacentor variabilis*) and soft (*Ornithodoros* spp.) ticks, mosquitoes (*Aedes albopictus*), sand flies (*Phlebotomus perniciosus*), poultry red mites (*Dermanyssus gallinae*) and sea lice (*Caligus rogercresseyi*) infestations and tick infection with *Anaplasma phagocytophilum, A. marginale*, *Babesia bigemina* and *Borrelia burgdorferi* [15, 19–21]. These results suggest that vaccines based on AKR/SUB antigens could control vector-borne diseases by a dual effect on vector populations and vector capacity [15, 16].

SUB/AKR intermediate proteins interactions with NF-kB and other regulatory proteins bind DNA and remodel chromatin to regulate gene expression of signal transduction and innate immune response genes and transcriptional regulators [16, 22–24]. This broad function of SUB/AKR as transcription factors explains the profound effect of gene knockdown by RNAi on tick and insect physiology, as well as on development and gene expression in ticks [15, 16]. SUB and AKR are functionally important for arthropod innate immunity and, at least in ticks, for tissue development and function and for pathogen infection and multiplication [16, 25].

A connection between the expression of mosquito AKR and *Plasmodium* spp. infection has not yet been established. However, several studies linked *Anopheles gambiae* NF-kB–like transcription factor REL2 to anti-parasitic defenses [26, 27]. These results together with the need to develop effective vaccines for malaria control have encouraged research on the possibility of using AKR for the control of *An. gambiae* populations and the infection with *Plasmodium* parasites.

The role of AKR on *Plasmodium berghei* infection and on the fitness and reproduction of the main malaria vector, *An. gambiae* was characterized by evaluating the effect of *akr* gene knockdown or vaccination with recombinant mosquito AKR on infection rate, parasite burdens, fertility and mortality of female mosquitoes.

## Methods

### Ethical statement

Animals were housed at the Instituto de Higiene e Medicina Tropical, in strict accordance with the recommendations of the Europe Directive 86/609/ EEC and Portuguese law (Decreto-Lei No. 129/92). Animal experiments were conducted with the approval of the Divisão Geral de Alimentação e Veterinária (DGAV), Portugal, under Art° 8, Portaria n°1005/92 from 23^rd^ October (permit number n° 023357). At the end of the experiment, mice were anesthetized before being euthanized by cervical disruption.

### Mosquitoes

The *An. gambiae s.s.* (molecular M form) of the Yaoundé strain mosquitoes were obtained from the Instituto de Higiene e Medicina Tropical (IHMT) insectary and reared at 26°C and 75% humidity on a 12/12 hour light/dark cycle. Adult mosquitoes were maintained on a 10% glucose solution.

### *Anopheles gambiae*infection with *Plasmodium berghei*

To obtain *An. gambiae* mosquitoes infected with *P. berghei* for gene expression and gene knockdown analyses, four weeks old female CD1 mice obtained from the IHMT animal house were intraperitoneally inoculated with 10^7^*P. berghei* parasitized red blood cells. GFP (PbGFPCON), a recombinant *P. berghei* strain that constitutively expresses GFP in the cytoplasm from a transgene controlled by the *elongation factor-1-alpha* gene promoter was used [28]. Parasitaemia were determined from blood samples collected from mouse tail, using light microscopy after methanol fixation of air-dried blood smears and staining with 10% (w/v) Giemsa. When the parasitaemia reached 10–20% and exflagellation was observed (4–6 exflagellations/field), mice were used to infect mosquitoes. Female mosquitoes (N = 200/mice) were allowed to feed directly on *P. berghei* infected mice (N = 3) for 30–45 minutes. Unfed female mosquitoes were removed from the cage. Fully engorged mosquitoes were kept at 19-21°C and 80% humidity for *P. berghei* development.

### Characterization of mosquito *akr*gene expression after parasite infection

The *P. berghei* infected and uninfected female mosquitoes were dissected. Midguts and the abdomen carcass (herein after denominated fat body) were stored in ice-cold RNA later (Ambion, Austin, TX, USA). Tissues were used immediately or stored at -20°C until RNA extraction. Pools of 30 infected/uninfected midguts and fat body were produced and total RNA was extracted using the Nucleospin RNAII kit (Macherey-Nagel, Bethlehem, USA) following the manufacturer’s instructions. First strand cDNA was synthesized using oligo dT and MMLV Reverse Transcriptase (Promega, Madison, USA). The *akirin* (Vectorbase: AGAP006809) expression levels were determined by qPCR using gene-specific oligonucleotide primers (5’- CCCTGTTCACCTTCAAGCAG-3’ and 5’- GGTCAGCACGGCATCATACT-3’) and iQ™ SYBR Green supermix in the iCycler iQ™ (Bio-Rad, Hercules, CA, USA) following the manufacturer’s recommendations. Fluorescence readings were taken at 62°C after each cycle and a melting curve was obtained to confirm the identity of the PCR product. Experiments were made in triplicate. The mRNA levels were normalized against ribosomal protein S7 gene (RPS7; Vectorbase: AGAP010592) using oligonucleotide primers (5’-GCCATCCTGGAGGATCTGGTA-3’ and 5’-CGATGGTGGTCTGCTGTTCTTATCC-3’) and the comparative ΔΔCt method [29]. Normalized mRNA levels were compared between infected and control mosquitoes by Student’s *t*-test with unequal variance (P = 0.05).

### Mosquito *akr*gene knockdown

RNAi was used to characterize the effect of *akr* gene knockdown in mosquitoes [30, 31]. The *akr* specific primers containing T7 promoter sequences at the 5’-end were synthesized (Table 1) and using the MEGAscript T7 kit (Ambion, Austin, TX, USA) dsRNA was produced according to manufacturer’s instructions. An exogenous gene, mouse *beta-2 microglobulin* (*B2m*) (GenBank: NM_009735) was used as control and the dsRNA was synthesized in a similar way (Table 1). The dsRNA was diluted in sterile water to a concentration of 3 mg/ml and concentration and quality were assessed by spectrometry and agarose gel. For gene knockdown, 600 female mosquitoes were used (200 mosquitoes/mouse). Cold anesthetized three day old-female mosquitoes were injected intrathoraxically with 69 nl of dsRNA using a nano-injector (Nanoject; Drummond Scientific, Broomall, PA, USA). The control group was injected with *B2m* dsRNA. Four days after dsRNA injection gene knockdown was analyzed by RT qPCR and the remaining mosquitoes were fed on a mouse infected with *P. berghei* and kept as above for parasite development. Surviving mosquitoes were counted and dissected 8 days after feeding. Midguts were mounted on coverslips and oocysts visualized under a fluorescent microscope to determine infection intensity (median number of parasite oocyst per infected mosquito) and the number of eggs in the ovaries. Infection rate (100 × [number of infected mosquitoes/total number of mosquitoes analysed]) was evaluated. Pools of approximately 30 infected/uninfected midguts and fat body were used to determine *akr* mRNA levels by RT-qPCR as described before. Normalized mRNA levels and the number of mosquitoes that survived dsRNA injection were compared between *akirin* dsRNA-injected and control mosquitoes injected with unrelated *B2m* dsRNA by Student’s *t*-test with unequal variance (P = 0.05). The number of parasite oocysts and eggs in mosquito midguts and the number of surviving mosquitoes were compared between *akr* dsRNA-injected and control mosquitoes injected with unrelated *B2m* dsRNA by a two-sample comparison using the non-parametric Mann–Whitney test (P = 0.0001).

**Table 1:** **Gene-specific primers and conditions used for dsRNA synthesis**

Gene (accession number)	Upstream/downstream primer sequence (5’-3’)	Fragment size (bp)	PCR annealing conditions
	TAATACGACTCACTATAGGGTACTTTGGCAGTCGTTGTAGTTGC		
Mosquito *akirin* (AGAP006809)		509	52°C/1 min
	TAATACGACTCACTATAGGGTACTCACCTGCTTGAAGGTGAACA		
	TAATACGACTCACTATAGGGAGACACCCCCACTGAGACTGATACA		
Mouse *B2m* (NM_009735)		447	62°C/45 sec
	TAATACGACTCACTATAGGGAGACACCCCCACTGAGACTGATACA		

T7 promoter sequences (5’-TAATACGACTCACTATAGGGTACT-3’) were included at the 5’-end for dsRNA synthesis.

### Mouse immunization and infection challenge

Recombinant AKR from *Ae. albopictus* was produced as previously reported using an extractive bioconversion process in an aqueous two-phase system supporting *Pichia pastoris* growth and protein secretion and used on immunization trials adjuvated in Montanide ISA 50 V2 (Seppic, Paris, France) [13, 17, 18]. Five weeks old female Balb/c mice (N = 5 per group) were obtained from the IHMT animal facility and used for the immunization trial. Mice were immunized intraperitoneally with 4 doses of 0.1 ml each containing 20 μg of recombinant AKR emulsified (1:1) with the adjuvant Montanide ISA 50 V2 (Seppic, Paris, France) [22] two weeks apart. Mice were immunized with AKR or adjuvant/saline and then infected with *P. berghei* or left uninfected as control. Two weeks after the last immunization, mice were infected with *P. berghei* by direct bite of infected mosquitoes. Thirty female mosquitoes, 3–5 days old, fed on each mouse for 30–45 minutes. After infective blood meal, only fully engorged females were transferred to individual vials for oviposition. Egg cups were removed seven days post-feed to count laid eggs per mosquito. Mosquito survival at 8 days post-infestation was also evaluated. Infection rate and infection intensity were determined as described before. Mosquito infection challenge was performed in three independent experiments. Results from mosquitoes fed on AKR-immunized and control mice were compared by Student’s *t*-test with unequal variance (P = 0.05).

### Determination of serum antibody levels by ELISA

Before each immunization and two weeks after the last immunization mouse tail blood was collected to prepare sera for analysis of antibodies titers against AKR by an indirect enzyme-linked immunosorbent assay (ELISA). A high binding 96 well-ELISA plate (Costar^®^, MA, USA) was incubated overnight at 4°C with 0.1 μg of recombinant protein per well diluted in 100 μl PBS. After antigen incubation, the plate was washed twice with tris buffered saline (25 mM Tris HCl, 150 mM NaCl, 2 mM KCl) containing 0.05% (v/v) Tween 20 (TBST), blocked with 200 μl of 5% (w/v) milk (BioRad, Hercules, CA, USA) at room temperature for one hour and washed three times with TBST. Serum samples were incubated for one hour at 37°C. The secondary anti-mouse AP-conjugated immunoglobulins (Sigma-Aldrich, St. Louis, Missouri, USA) were diluted 1:10,000 in TBST supplemented with 0.1% (w/v) BSA (Sigma-Aldrich, St. Louis, Missouri, USA), added to wells and incubated for one hour at 37°C. After washing 5 times with TBST, plates were incubated with 1 mg/ml of p-nitrophenil phosphate in substrate buffer (100 mM glycine, 1 mM MgCl_2_, 1 mM ZnCl_2_, pH 10.4) at room temperature in the dark. To stop the reaction, 100 μL of 3 M NaOH was added to each well. Plates were then red in a microplate reader (BioRad model 550) at a wavelength of 405 nm and analyzed with the Microplate manager 4.0 software (BioRad). Antibody titers were determined at a 1:6,400 serum dilution and were compared between AKR-immunized and control mice by Student’s *t*-test with unequal variance (P = 0.0001).

## Results

### The *akr*gene knockdown resulted in reduced mosquito survival and egg production and increased parasite infection

For the characterization of *akr* gene expression in response to parasite infection, adult female mosquitoes were infected with *P. berghei*. Mice were infected with *P. berghei* or left uninfected as control. Twenty-four hours post-infection, adult female mosquitoes were fed on mice to characterize *akr* expression in midguts and fat body tissues. To characterize the *in vivo* effect of *akr* knockdown by RNAi on mosquito biology and parasite infection, injection of *akr* and unrelated *B2m* control dsRNAs was performed in 600 female mosquitoes fed on 3 different infected mice (200 mosquitoes/mouse). The *akr* gene mRNA levels were significantly lower in *akr* dsRNA-injected mosquitoes when compared to dsRNA controls (P < 0.05) with gene silencing ranging from 16-40% in the midgut and 25-65% in the remaining tissues.

**Figure 1:**
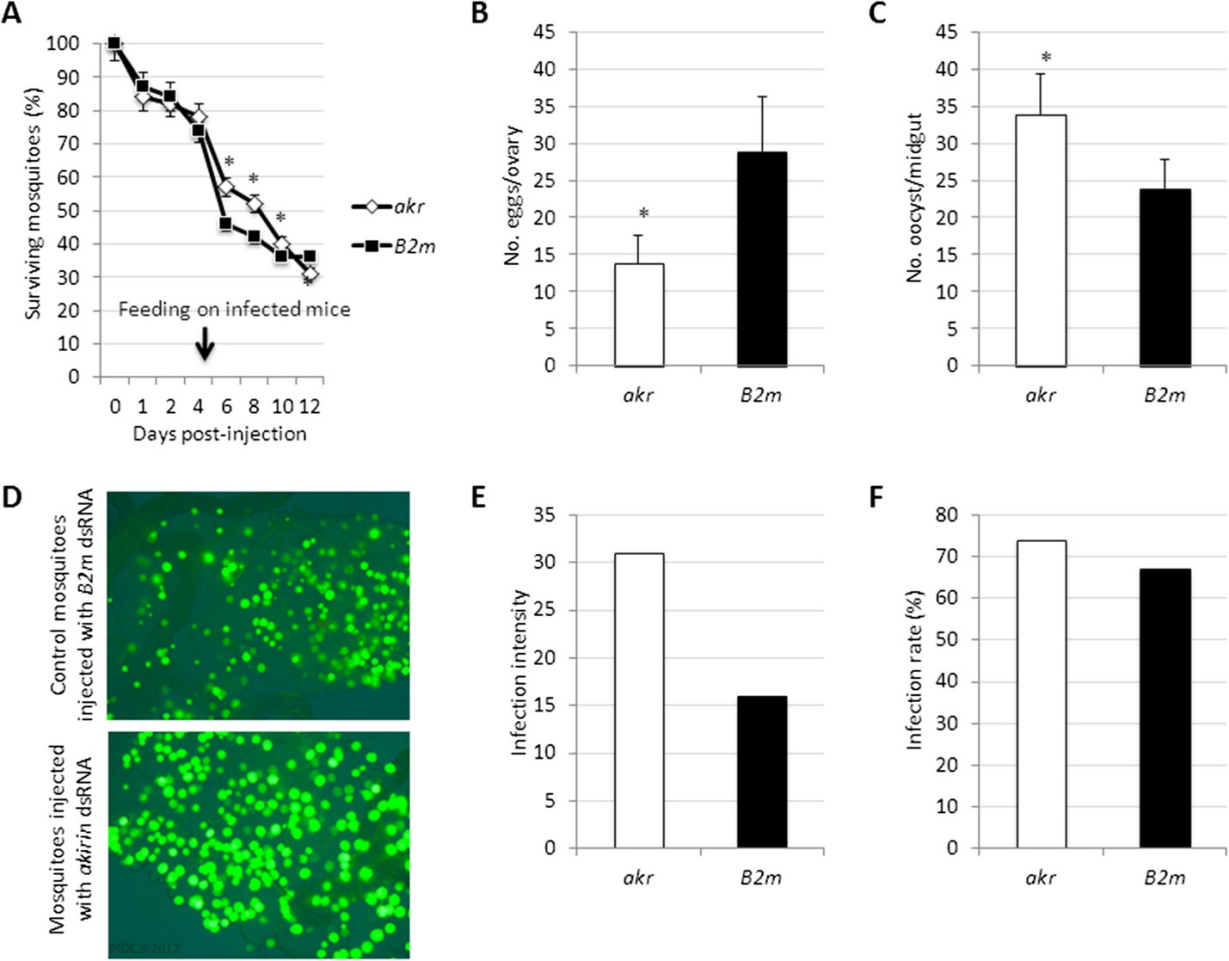
**Effect of mosquito** ***akr*** **gene knockdown.** Mosquitoes (N = 3 experiments of 200 mosquitoes each) were injected with dsRNA and fed 4 days later on *P. berghei*–infected mice. Surviving mosquitoes were counted and dissected to collect midguts 8 days after feeding (day 12 post-injection) to determine infection intensity (median number of parasite oocyst per infected mosquito), infection rate (100 × [number of infected mosquitoes/total number of mosquitoes analyzed]), number of parasite oocyst in mosquito midguts, number of eggs in the ovaries and the number of surviving mosquitoes**. (A)** Surviving mosquitoes. **(B)** Number of eggs per ovary. **(C)** Number of oocyst per midgut. **(D)** Representative fluorescence images of parasite oocyst in mosquito midguts. **(E)** Representative results for infection intensity. **(F)** Representative results for infection rate. Similar results were obtained in all replicates (N = 3). The number of parasite oocyst/midgut and eggs/ovary and the number of surviving mosquitoes (Ave ± SD) were compared between *akr* dsRNA-injected and control mosquitoes injected with unrelated *B2m* dsRNA by a two-sample comparison using the non-parametric Mann–Whitney test (*P < 0.0001).

The effect of *akr* gene knockdown on mosquito mortality, egg production and *P. berghei* infection were characterized. Mosquito survival, evaluated six days after infectious blood meal, was higher in *akr* dsRNA-injected mosquitoes when compared to controls (Figure 1A). However, at day-8 post-infectious blood feeding (day 12 post-injection), survival was 14% lower in mosquitoes with *akr* gene knockdown (Figure 1A). The *akr* gene knockdown also reduced egg production by 52% when compared to control mosquitoes injected with *B2m* dsRNA (Figure 1B). However, parasite infection, determined by different parameters was higher in mosquitoes with *akr* knockdown when compared to controls (Figures 1C-F).

### Anti-AKR antibodies reduced *P. berghei*infection in *An. gambiae*fed on immunized mice

**Figure 2:**
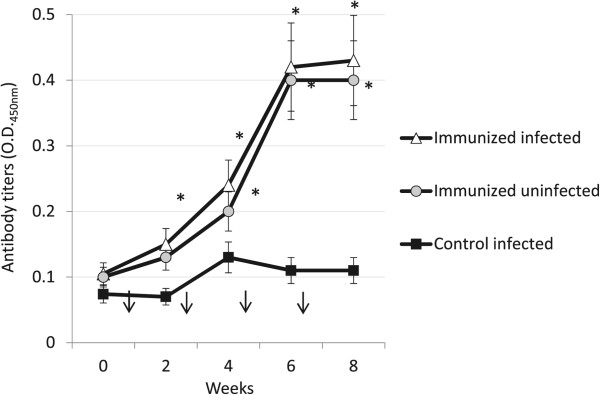
**Mice immunization with AKR.** Antibody titers were determined by ELISA and compared between AKR-immunized and control mice by Student’s *t*-test with unequal variance (*P < 0.0001). Immunization shots are represented with arrows.

**Figure 3:**
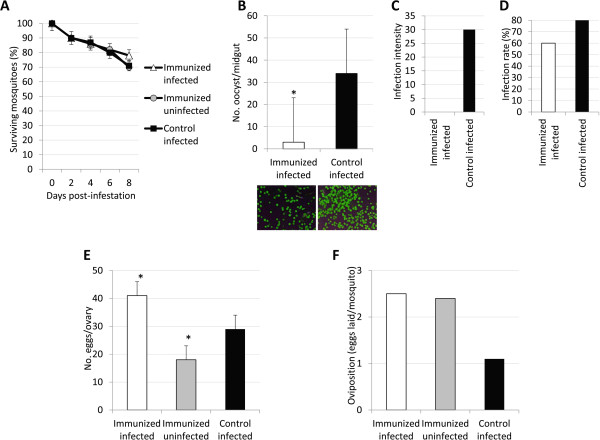
**Effect of immunization with AKR on mosquito biology and infection with** ***P. berghei.*** Mice were immunized with recombinant AKR or adjuvant/saline and then infected with *P. berghei* or left uninfected as controls. **(A)** Surviving mosquitoes. **(B)** Number of oocyst per midgut with representative fluorescence images of parasite oocyst in mosquito midguts. **(C)** Representative results for infection intensity. Similar results were obtained in all replicates (N = 5). **(D)** Representative results for infection rate. Similar results were obtained in all replicates (N = 5). **(E)** Number of eggs per ovary. **(F)** Oviposition (representative results for the number of laid eggs/mosquito; similar results were obtained in all replicates; N = 5). The number of parasite oocyst/midgut, eggs/ovary and the number of surviving mosquitoes (Ave ± SD) were compared between mosquitoes fed on immunized and control infected mice by a two-sample comparison using the non-parametric Mann–Whitney test (*P < 0.0001).

To evaluate the effect of antibodies against AKR on the malaria vector *An. gambiae* and the infection with *P. berghei*, mice were immunized with recombinant mosquito AKR or placebo and infected with *P. berghei* parasites or left uninfected as controls. Mouse antibody titers increased after the first immunization with recombinant AKR and remained significantly higher until the end of the experiment in both AKR-immunized infected and uninfected mice when compared to controls (Figure 2). Different to *akr* knockdown, survival was not affected in mosquitoes fed on immunized mice when compared to mosquitoes fed on control mice (Figure 3A). Also in contrast to RNAi results, parasite infection was lower (Figures 3B-D) and egg production was higher (Figures 2E and F) in mosquitoes fed on immunized infected mice when compared to mosquitoes fed on control infected mice. Particularly relevant was the effect on infection intensity, which was reduced in more than 60-fold in mosquitoes fed on immunized mice when compared to controls (Figure 2C). Egg production was significantly lower in mosquitoes fed on immunized uninfected mice when compared to mosquitoes fed on control infected mice (Figure 2E).

## Discussion

The WHO estimates that 207 million cases of malaria and 627,000 deaths occurred globally in 2012. The mosquito *An. gambiae* is the major vector of parasites causing malaria in sub-Saharan Africa [2]. The increasing prevalence of malaria is attributed to the rapid spread of drug-resistant parasites, insecticide-resistant mosquitoes and the absence of a protective vaccine. Our studies were focused on understanding the role of mosquito AKR, a conserved nuclear factor required for innate immune responses, in the complex interactions between the vector and *Plasmodium* parasite and the potential of this protein as a protective vaccine antigen. As previously shown, tick SUB and mosquito AKR are good protective antigen candidates for the control of both vector infestations and pathogen infection [15, 16, 25].

RNA interference-based *akr* knockdown in mosquitoes was performed as previously reported for other target genes [32]. Obtained data suggests that, as in prior experiments concerning to *akr/sub* RNAi assays in flies [22] and ticks [33–36], mosquito survival and egg production were reduced after gene knockdown, supporting the role of AKR in immune response. RNAi mediated knockdown led to an increase of parasite infection in *An. gambiae* mosquitos after feeding on infected mice as reported for *akr* knockdown in flies infected with *Agrobacterium tumefasciens* [22]. Also in white shrimps (*Litopenaeus vannamei*) *akr* mRNA levels are strongly induced in response to *Vibrio parahaemolyticus* infection and *akr* silencing lead to high mortality after infection challenge [37]. The effect of *Akr* silencing on infection level is probably due to the impair of Imd pathway signaling enhancing the sensitivity to bacterial and parasite infection [15, 16, 22, 25]. The increase of *Plasmodium* parasites levels after gene silencing shows that AKR/SUB is required for defense against pathogens and for the regulation of genes that are important for infection.

The AKR gene regulation function makes it a potential antigen for vaccine development against vector infestations. Thus, to determine whether AKR could represent a novel target for an *An. gambiae* infestations control and also to determine its influence on *Plasmodium* infection process, we vaccinated mice with recombinant AKR from *Ae. albopictus*.

Following silencing data, AKR vaccination was expected to primarily reduce *An. gambiae* reproductive and survival parameters since it has been reported that this protein is related not only to the embryonic development of insects [38] or involved in muscle development [39] but also influences infection levels due to its immunity related functions. Previous immunization experiments using this antigen showed a reduction in the oviposition and fertility of *Ae. albopictus* mosquitoes fed on immunized uninfected mice when compared to controls [17, 18]. Egg production was also reduced in *An. gambiae* fed on AKR immunized uninfected mice but AKR did not affect *An. gambiae* survival after feeding on immunized mice in accordance to other reports [17, 18].

Antibodies against recombinant AKR significantly reduced the infection on *An. gambiae* by *P. berghei* suggesting that AKR might contribute to the development of vaccines against malaria by reducing parasite infection levels in vector mosquito species. In opposition of what was expected, results suggest that blocking the AKR protein in the cells did not distress Imd pathway but rather we may be targeting protein translocation disturbing, thus, the cell response to infection [25].

The protection elicited by the anti-mosquito vaccine is based on the production antibodies in the vaccinated mice that may interact and affect the function of the target antigen (AKR) leading to a response against *Plasmodium* proteins. The development of vaccines against blood-sucking arthropod ectoparasites such as mosquitoes is based on the concept that ectoparasites ingest antigen-specific antibodies when feeding on immunized hosts. These antibodies interact with the protective antigen in the arthropod to affect the function and/or quantity of the protein, resulting in reduced survival, reproduction and/or infection with vector-borne pathogens. For SUB/AKR, the mode of action of the antibodies is not completely understood but may be the result of antibodies crossing the cell membrane and interacting with the protein in the cytoplasm to prevent protein translocation to the nucleus [15, 16, 25, 35, 40]. The issue of vaccine safety with antigens such as AKR that are conserved between invertebrate and vertebrate hosts has been addressed before, suggesting in a low risk to induce autoimmune responses in vertebrate hosts [17, 18, 33].

## Conclusions

In summary, the results reported here showed that recombinant AKR could be used to develop vaccines for malaria control. If effective, AKR-based vaccines could be used to immunize wildlife reservoir hosts and/or humans to reduce the risk of pathogen transmission.
